# Candidates for Admission into the Army

**Published:** 1840

**Authors:** 


### CANDIDATES FOR ADMISSION INTO THE MEDICAL STAFF OF THE ARMY. MEDICAL SCHOOLS.

From the last Report of the Surgeon General, of the United States, to the Secretary at War, we make the following extracts:

"The law requiring an examination of candidates for appointment and of assistant surgeons for promotion in the medical department of the army, has been rigidly enforced.

"Two junior surgeons whose examination for promotion had been unavoidably deferred, and three assistant surgeons of five years standing, were ordered to present themselves, and thirty-six applicants for appointment to the medical staff of the army, were invited to appear before the medical board lately in session, at New York. The surgeons and assistant surgeons having undergone a thorough examination upon all the branches of medical science, and received a favourable report from the board, the first two were sustained in their advanced position, and the last three rendered *legally* qualified for promotion. With the candidates for admission into the army, however, the result of the examination was very different. Of the thirty-six who were invited to appear before the board, twelve declined the examination, (two, after having reported to the the board,) two were excluded on account of their age, and twenty-two were examined; and of these last, five only were found to possess all the qualifications essential to an appointment.

"It may be that we have erected too high a standard of merit—that too much is exacted from the human intellect; we are not conscious, however, that more has been asked, than ordinary talents, a good primary education, and the actual study of the science of medicine, can attain. At all events, some few have reached the highest scale of excellence; and while as many of these choice spirits can thus be secured, as will fill our ranks in each succeeding year, we shall not relax in our requirements upon those who claim to be admitted into the medical staff of the army.

"But to account for the humiliating result of the examination on the present and on the former occasions, we have only to look to the system of education which now obtains in the country.

"The facilities of acquiring medical knowledge, or rather of becoming professional men, are so great, that many persons are seduced into an attempt to become physicians, without the basis of an education. There are others again, who having received a good primary education, and also passed through a regular classical or collegiate course, (and thereby rendered qualified for scientific pursuits,) are induced from motives of economy and convenience, or with the view of sustaining institutions of their own State, to enter some of the small medical schools, where they cannot possibly have the advantages of anatomical dissection, (the ground work of the profession,) or the means of clinical instructions upon an extended scale. A knowledge of the science of medicine is not, like divinity and law, to be acquired by reading books in the closet, and listening to the reading of a course or two of lectures; it can only be attained by seeing and feeling, in connection with the knowledge acquired from books.

"The great multiplication of medical schools in every section of the country, together with the proverbial facilities of becoming licensed practitioners, has so lowered the standard of professional excellence, and so manifestly degraded the medical character of the United States, that the present system will be, it is to be hoped, by a more enlightened public opinion ere long put down. The interests of the country are so much divided by these various institutions, and the patronage to each is consequently so small, that many of our ablest medical men will not accept places in them; were it practicable, however, for the professors to obtain adequate compensation for their services, it would be impossible to find professional men enough of talents and attainments to occupy the several chairs in the innumerable medical schools in every town, village, and cross-road place, throughout our states and territories."

There is much matter for serious, indeed painful, reflection in these extracts. Out of twenty-two candidates, all we presume graduates, five only, not a fourth, were found qualified! Whence this discrepancy between the Faculties of our schools and the Medical Board of the Army? Are those who are qualified to practise among citizens, unfit to practise among soldiers? In a time of general war, this, from the special demand which would then exist for surgical skill, might in some degree be the case; but such a war does not, now, exist, and indeed the report does not intimate, that the rejected candidates, were deficient in surgery more than physic. We may presume then, that they failed on the ground of general professional incompetence; and if so, it is manifest that the Army Board have erected a higher standard, than the schools, in which the seventeen rejected applicants were graduated. Which is in the right? We know not from what schools those gentlemen received their diplomas, but although teachers ourselves, we are bound to admit, that the rejections *may* have been justly and properly made; from which admission it follows, that we think the standard of our schools too low—that of the army not too high.

That many young gentlemen receive diplomas who ought not to receive them, and who could not obtain them from any, even the lowest, schools in Europe, we have long been convinced. Were this true only of the alumni of the more obscure, and what may be called, provincial institutions, it would be of less moment; but ample and long continued observation has assured us, that the oldest and most celebrated establishments of the Union, are in this respect as great sinners as the humblest. This being the case, we regard with satisfaction the efforts of the Army Board to apply a corrective, and hope to see them persevere. We are far, however, from believing, that they alone can cure the evil, or that they have judged correctly as to its cause, when they fix upon the "multiplication of medical schools in every section of the country." Of this multiplication the Report speaks, with a flippancy unworthy of the subject. We admit that many of our schools have in them unqualified teachers, but we do not admit that the graduates of these schools, are particularly inferior to those educated under abler men; nor do we admit that talented teachers in sufficient numbers, could not be found. The Surgeon General thinks that the multiplication of medical schools increases the number of medical students. We believe that it increases the number who attend lectures, but does not increase the number of youth who engage in the study of medicine. The foundations of the difficulty lie deeper than the report has penetrated.

*First.* The compensation of medical men generally, throughout the Union, is such as to repel, rather than invite young men of talent, education, and enterprise, into the ranks of the profession; especially, when commerce and the law, hold out much higher inducements.

*Second.* As we have intimated in another article of this number, the sessions of our schools are too short, and cannot be lengthened but by common consent, which consent however could undoubtedly be obtained by a proper effort.

Our short sessions explain why there is less difference, than might be expected, between the graduates of the most distinguished and the most humble institutions of the country. The time allotted for a sojourn within their walls, is too short to admit of any attainment beyond the *rudiments* of the science, which are often as successfully imparted, by men of limited knowledge, as by the most erudite.

*Third.* It is in vain to declaim against the multiplication of medical schools. At present they about equal the number of States; but they are unequally divided among these rival Commonwealths; and, under the great principle of emulation, we may expect, that several which have not yet chartered medical schools will hereafter do it.

The true remedy then is, a leghtened session, and greater liberality of compensation to our physicians generally. D.

